# Mitochondrial genome analysis, phylogeny and divergence time evaluation of *Strixaluco* (Aves, Strigiformes, Strigidae)

**DOI:** 10.3897/BDJ.11.e101942

**Published:** 2023-03-20

**Authors:** Yeying Wang, Haofeng Zhan, Yu Zhang, Zhengmin Long, Xiaofei Yang

**Affiliations:** 1 Guizhou Normal University, Guiyang, China Guizhou Normal University Guiyang China; 2 Guizhou University, Guiyang, China Guizhou University Guiyang China

**Keywords:** *Strixaluco*, phylogeny, divergence time, Pleistocene, climate oscillation, mountains uplift

## Abstract

Background

Prior research has shown that the European peninsulas were the main sources of *Strixaluco* colonisation of Northern Europe during the late glacial period. However, the phylogenetic relationship and the divergence time between *S.aluco* from Leigong Mountain Nature Reserve, Guizhou Province, China and the Strigiformes from overseas remains unclear. The mitochondrial genome structure of birds is a covalent double-chain loop structure that is highly conserved and, thus, suitable for phylogenetic analysis. This study examined the phylogenetic relationship and divergence time of *Strix* using the whole mitochondrial genome of *S.aluco*.

New information

In this study, the complete mitochondrial genome of *Strixaluco*, with a total length of 18,632 bp, is reported for the first time. A total of 37 genes were found, including 22 tRNAs, two rRNAs, 13 protein-coding genes and two non-coding control regions. Certain species of Tytoninae were used as out-group and PhyloSuite software was applied to build the ML-tree and BI-tree of Strigiformes. Finally, the divergence time tree was constructed using BEAST 2.6.7 software and the age of *Miosurniadiurna* fossil-bearing sediments (6.0–9.5 Ma) was set as internal correction point. The common ancestor of *Strix* was confirmed to have diverged during the Pleistocene (2.58–0.01 Ma). The combined action of the dramatic uplift of the Qinling Mountains in the Middle Pleistocene and the climate oscillation of the Pleistocene caused *Strix* divergence between the northern and southern parts of mainland China. The isolation of glacial-interglacial rotation and glacier refuge was the main reason for the divergence of *Strixuralensis* and *S.aluco* from their common ancestor during this period. This study provides a reference for the evolutionary history of *S.aluco*.

## Introduction

*Strixaluco* belongs to Strigidae (Strigiformes) and is a medium-sized owl (*Grytsyshina et al. 2016*). *S.aluco* is a non-migratory and territorial nocturnal bird (Sunde et al. 2003, Doña et al. 2016) with a wide distribution throughout mountainous broadleaf forest and mixed forest in Eurasia; Israel is the southernmost country in the distribution area of *S.aluco* in the Northern Hemisphere (Obuch 2011, Comay et al. 2022). It can feed on mammals, fish, amphibians and even small birds such as sparrows (*Obuch 2011*) and voles are its most preferred food (Karell et al. 2009, Solonen 2022). The IUCN listed this species as of "Least Concern". The current population trend is stable and the estimated number of individuals ranges from 1,000,000 to 2,999,999 (IUCN 2016: https://www.iucnredlist.org/). In China, *S.aluco* has been listed as a national class II protected animal.

Mitochondria are characterised by maternal inheritance, high conservation, multiple copies in each cell, low sequence recombination rate and high evolutionary rate; therefore, mitochondria are widely used in phylogenetic studies (Yan et al. 2017, Sun et al. 2020). Using them enables researchers to accurately infer phylogenetic relationships in birds (Tuinen et al. 2000) and complete mitochondrial genomes generally achieve higher accuracy than partial mitochondrial genomes (Haring et al. 2001, Harrison et al. 2004). Through skeletal comparison, Strigidae has been divided into three subfamilies: Striginae (13 genera), Surniinae (eight genera) and Asioninae (two genera) (Ford 1967). Previous studies have defined the phylogenetic position of *S.aluco* using a single gene or a combination of multiple mitochondrial genes (Heidrich and Wink 1994, Fuchs et al. 2008, Wood et al. 2017, Yu et al. 2021). Earlier research identified the monophyly of the Strigiformes phylogeny through the cytochrome B (Cyt B) gene (Wink and Heidrich 2000). In Strigiformes, the taxonomic relationship of subordinate branches of Strigidae has been hotly debated (Salter et al. 2020). Phylogenetic relationships through the Cyt B gene also showed that the order Strigiformes can be divided into two groups: (Tytonidae + Strigidae), Tytonidae consisting of Tytoninae (containing *Tyto*) and Phodilinae (containing *Phodilus*); and Strigidae can be divided into Striginae, Surniinae and Ninoxinae, amongst them, Striginae can be subdivided into a clade of ((*Bubo* + *Strix*) + (*Pulsatrigini* + *Asio*)) + (*Psiloscops* + *Megascops*) + *Otus*, with Surniinae consisting of two branches (with Surniini and *Aegolius*), Surniini containing (*Glaucldium* + *Athene*) and Ninoxinae being mainly composed of *Ninox*, possibly including *Uroglaux* and *Sceloglaux* (Wink et al. 2009, Wink and Sauer-G&uuml 2021). In addition, a growing number of scholars have described a framework for Strigiformes phylogeny. Zhang et al. (2016) completed the whole mitochondrial genome sequencing of *Asioflammeus* and determined the paraphyletic phylogenetic relationship amongst the three genera of *Otus*, *Ptilopsis* and *Asio*; Kang et al. (2018) completed the whole mitochondrial genome sequencing of *Strixuralensis* and determined the inter-genus relationship of *Otus* + (*Asio* + (*Strix* + *Bubo*)) by studying the mitochondrial genome of Strigidae; Salter et al. (2020) combined morphological characteristics and molecular biology, suggesting that a typical owl contains Striginae and Surniinae; they further suggested that *Athene*, *Otus*, *Asio*, *Megascops*, *Bubo* and *Strix* are paraphyletic, while *Ninox* and *Glaucidium* are polyphyletic; Koparde et al. (2018) showed that the Striginae and Surniinae form a paraphyletic group in the South Asian subcontinent population with Tytonidae as the out-group; their study showed that Strigidae and Tytonidae diverged at about 42.5–47.7 Ma (mega-annum, million years); Uva et al. (2018) clarified the global distribution of Tytonidae and their time of divergence, their analysis showing that Tytonidae and *S.aluco* split from a common ancestor dating back to about 45 Ma. Prior research has shown that *Strix* and *Tyto* diverged roughly about 40–50 Ma (Prum et al. 2015). The phylogenetic relationship and timing of the divergence of *Strix* in China remain unclear.

There are many reasons for the divergence of species, amongst which geological and climatic influences on species diversification cannot be ignored (Claramunt and Cracraft 2015). The Cretacean-Tertiary extinction event was a mass extinction event in Earth's history that occurred 65 Ma and wiped out most animals and plants at the time, including the dinosaurs. It also wiped out the direct ancestors of tree-dwelling waterbirds on Earth today with the few survivors evolving rapidly thereafter (Field et al. 2018). Bird ancestry began to increase exponentially at the end of the Eocene, from an original 100 species to the 10,000 species of today (Ksepka and Phillips 2015). Since the late Miocene, many birds in the Palaearctic have migrated on a large scale and their changing ranges have led to gene flows that have provided opportunities for the origin of various bird subfamilies (Drovetski 2003, Holm and Svenning 2014). Climatic oscillation during the Quaternary Period, especially throughout the Pleistocene (2.58–0.01 Ma), promoted the evolution of species on a global scale (Hewitt 2000, Hewitt 2004, Lamb et al. 2019). Pleistocene glacial gyre played a positive role in speciation (Zhao et al. 2013, Hung et al. 2014, Kozma et al. 2018, Mays et al. 2018). Brito (2005) studied 14 populations of *S.aluco* in Western Europe and found that these could be divided into three branches originating from three glacial sanctuaries in the Iberian Peninsula and the Balkan Peninsula in Europe. This finding supports the "glacier refuge hypothesis" that describes the origin of *S.aluco* in estern Europe. However, the origin and divergence of *S.aluco* in mainland China remain a mystery.

Divergence time analysis can provide a reference for the evolution process of species and provides a basis for further studies. To clarify the divergence time of species, it is necessary to obtain their gene sequence first; then, an appropriate evolutionary model needs to be selected and reliably calibrated, for example, by determining the age of fossils (Ho and Phillips 2009, Ho and Duchêne 2014). To clarify the phylogenetic position, divergence time and reasons for divergence of *S.aluco* from China, this study sequenced the complete mitochondrial genome of *S.aluco* and used it (combined with the mitochondrial genome of other birds in Strigiformes) to reconstruct the phylogenetic tree of Strigiformes. Fossil data are usually used to evaluate the divergence time of birds and the divergence time of Surniinae fossils was used as the correction point to analyse the divergence time of *Strix*. Possible reasons for its divergence are discussed in depth.

## Materials and methods

### Sample origin and DNA extraction

Part of the muscle tissue was extracted from the leg of one individual of *S.aluco* that died of an unknown cause in the Rescue Center of Leigong Mountain National Nature Reserve, Qiandongnan Prefecture, Guizhou Province, China (26° 49' 26.40" N, 104° 43' 33.60" E). The sample was stored in a refrigerated box with a built-in thermometer, the temperature was kept near freezing, until the sample was transported back to the laboratory for DNA extraction. To extract DNA, the standardised CTAB method was used (Lutz et al. 2011).

### Sequencing and assembly

The whole genome shotgun strategy was used to construct the library (*Roe 2004*). Next generation sequencing technology was used for paired-end sequencing, based on the Illumina NovaSeq sequencing platform (Illumina NovaSeq, Illumina Inc., San Diego, California, USA).

The concentration and purity of DNA extracted from the samples were assessed by Thermo Scientific NanoDrop 2000 (Thermo Scientific NanoDrop 2000, Thermo Fisher, Massachusetts, USA) and the integrity was assessed by agarose electrophoresis (Electrophoresis apparatus of Liuyi Company, Beijing, China) and Agilent 2100 Bioanalyzer (Agilent 2100 Bioanalyzer, Agilent Corporation, California, USA). The Covairs machine (Covairs machine of BRANSON Company in St. Louis, Missouri, USA) was used to break up and fragment DNA. The gene library was constructed according to the shotgun method described by *Roe (2004)*. The Agilent 2100 Bioanalyzer was used to assess the size of the library and fluorescence quantitative detection was used to assess the total concentration of the library. The optimal amount of the library was selected and sequenced on the Illumina NovaSeq sequencing platform. A single-stranded library was used as a template for bridge PCR amplification and sequencing was performed during synthesis.

After DNA extraction, purification, library construction and sequencing, a raw image file was first obtained by sequencing. The raw data that can be read in FASTQ format were generated after the multi-step transformation, i.e. the offline data. Data transformation is automatically completed by the sequencing platform. According to the statistics of raw data, 7,947,240 reads (each sequence read is called one read) were obtained, the total number of bases was 1,192,086,000 bp, the percentage of fuzzy bases (uncertain bases) was 0.0016% and the GC content was 44.58%. The base recognition accuracy exceeding 99.00% accounted for 95.61% and the base recognition accuracy exceeding 99.90% accounted for 90.44%. The quality of off-machine data was tested through quality control and the software used is FastQC (http://www.bioinformatics.babraham.ac.uk/projects/fastqc).

Sequencing data contain low-quality reads with connectors, which will greatly interfere with subsequent analysis. To ensure the quality of subsequent information analysis, Fastp software (version 0.20.0) was used to remove sequencing connectors at the 3' end. Low-quality sequences (i.e. sequences with an average Q value of less than 20 and sequences with a sequence length shorter than 50 bp) were removed. The number of high-quality reads obtained was 7,611,984, accounting for 95.78% of the raw data and the number of bases of high-quality reads was 1,123,739,765 bp, accounting for 94.27% of the raw data (Chen et al. 2018).

A5-miseq v20150522 (Coil et al. 2015) and SPAdesv 3.9.0 (Bankevich et al. 2012) were used for the *de novo* sequencing of high-quality next-generation sequencing data. To construct contig and scaffold sequences, the sequences were extracted according to the sequencing depth of *de novo* splicing sequences. The sequences with high sequencing depth were compared with the NT (Nucleotide) library on NCBI by Blastn (BLAST v2.2.31+) and the mitochondrial sequences of each splicing result were selected. To integrate splicing results, the mitochondrial splicing results obtained by the different software above were combined with reference sequences. Collinearity analysis was performed using mummer v.3.1 software (Kurtz et al. 2004) to determine the position relationship between contigs and fill gaps between contigs. The results were corrected by using pilon v.1.18 software (Walker et al. 2014) to obtain the final mitochondrial sequence. The complete mitochondrial genome sequence obtained by splicing was uploaded to the MITOS web server (http://mitos2.bioinf.uni-leipzig.de/index.py) for functional annotation (Bernt et al. 2013). RefSeq 81 Metazoa was selected as reference, the genetic code was set to a second set of vertebrate codons and other parameters were set according to the default parameters proposed by MITOS.

Through the above methods, the base compositions of the whole mitochondrial genome, protein-coding genes and rRNA genes were obtained. CGview visualisation software was used to draw the mitochondrial complete genome circle map (Stothard and Wishart 2005).

### Mitochondrial genome data collection in Strigiformes

Currently (until this study), in GenBank, there are 30 species with mitochondrial genomes greater than 10,000 bp, including 27 species of Strigidae and three species of Tytonidae. All taxonomic classifications of the species follow the current version of the IOC WORLD BIRD LIST (12.2) (http://dx.doi.org/10.14344/IOC.ML.12.2). The existing sequences of these thirty species were stored in a local folder using GenBank format. The registration number is shown in Table 1.

### Construction of phylogenetic trees

The PhyloSuite software (downloaded from: https://github.com/dongzhang0725/PhyloSuite/releases) (*Zhang et al. 2020*) was used to drag the 30 GenBank format files (downloaded from NCBI) and the GenBank format files of *S.aluco* sequence (obtained by the sequencing in this study) into the main interface.

First, following the guided steps in the literature of Zhang et al. (2020), a series of standardised operations were conducted. Mitogenome sequence types were chosen; meanwhile, the annotation error tRNA file was exported and modified comments were uploaded to the ARWEN website (http://130.235.244.92/ARWEN/). The site of the modified comments is copied and pasted for modification after the box. After that, the corrected 13 protein-coding genes (PCGs) and 24 RNAs were extracted successfully. The second set of two vertebrate mitochondrial codes is selected here and the extracted 13 PCGs and 24 RNAs are imported into MAFFT (PhyloSuite programme) for multiple sequence alignment. The 37 gene files exported by MAFFT were selected and imported into 'concatenate sequence' (PhyloSuite programme), using the '-auto' strategy and 'normal' alignment mode. The concatenated completion file was selected and PartitionFinder 2.0 (PhyloSuite programme) (*Lanfear et al. 2017*) was used to perform a greedy search, select the 'Nucleitide' mode and 'branc-lengths' can be 'linked'. Here, 'mrbayes' was chosen for models and the model supported by MrBayes was calculated. 'AICc' is the model selection criterion recommended by PartitionFinder authors, the optimal partitioning strategy and model selection was calculated, this place using a separate GTR+G model for each data block automatically.

The result file of PartitionFinder 2.0 was selected, the ML method was completed in IQ-tree (Minh et al. 2020) mode (PhyloSuite programme) and the BI method was completed in MrBayes mode (PhyloSuite programme). *Phodilusbadius*, *Tytoalba* and *Tytolongimembris* were set as out-groups. In IQ-tree mode, the Edge-linked partition style was employed for 10,000 replicates of ultrafast bootstrapping (Sterli et al. 2013, Hoang et al. 2018). In MrBayes mode, the result folder of PartitionFinder 2.0 was opened, out-groups were set, parameters were defined as Partition Models and algebra run as two parallel runs, four chains, for 2,000,000 generations (where it must be ensured that the average standard deviation of split frequencies values remains below 0.01), sampling freq is one sampling run for 1000 times and 25% of the initial samples were discarded as burn-in.

### Divergence time evaluation

*Miosurniadiurna* fossils provide an approximate date of the origin of Surniinae and the age of the fossil-bearing sediments of the *M.diurna* is 6.0–9.5 Ma (*Li et al. 2022*), the origin times of Surniinae were set to 6.0 Ma and 9.5 Ma, which included *Aegolius*, *Athene*, and *Glaucidium* (*Wink and Sauer-G&uuml 2021*). The 'NEX' file obtained by concatenating 37 genes using the 'concatenate sequence' programme function in PhyloSuite was imported into BEAUti 2.6.7, (http://www.beast2.org/), Hasegawa-Kishino-Yano model, with four gamma categories, Strict clock with 1.0 clock rate and with a Yule process (speciation) prior. “*Aegoliusfunereus*, *Athenebrama*, *Athenenoctua*, *Glaucidiumbrasilianum*, *Glaucidiumbrodieibrodiei* and *Glaucidiumcuculoides*” (sequence file name) was chosen and Prior was added. Then, the “monophyletic” option was checked, the Mean set to 6.0/9.5 and Sigma set to 0.1. A Markov Monte Carlo Chain Bayesian analysis with a chain length of 10,000,000 and with states recorded every 1000 iterations was run using BEAST 2.6.7. Log files were assessed using TRACER 1.7.2 (http://tree.bio.ed.ac.uk/software/tracer/) to ensure that posteriors were normally distributed and that all statistics had attained effective sample sizes of > 200. If ESS < 200, optimisation was employed by adding 5,000,000 iterations (chain length) each time. A burn-in of 10% was discarded, the maximum clade credibility tree was determined and mean heights were chosen using TreeAnnotator 2.6.7. Finally, FigTree 1.4.4 was used to assess the divergence time. Finally, Adobe Illustrator 1.0.0.2 was used for visual editing (all figures are the same).

## Results

### Genome annotation

**Figure 1.**
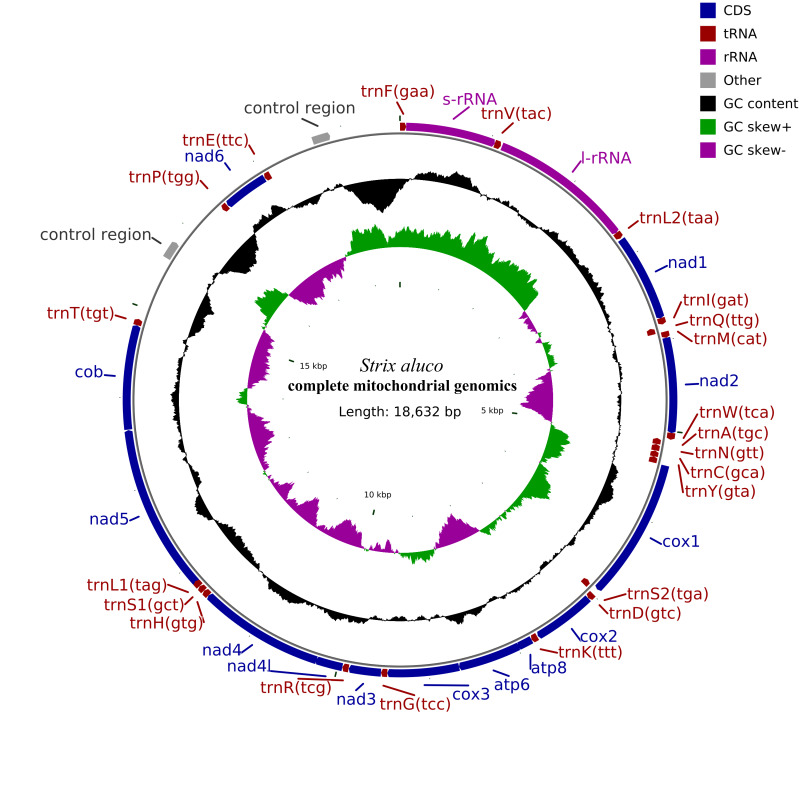
Complete mitochondrial genome of *S.aluco*. The total length of the mitochondrial genome of *S.aluco* was 18,632bp. The genes located on the N strand or J strand are positioned inside or outside the circle. Contains two D-Loop regions. The GC Skew+ region contains more Guanine than Cytosine and the GC Skew- region contains more Cytosine than Guanine.

The total length of the mitochondrial genome sequence was 18,632 bp (GenBank entry number: OP850567). The results of genome annotation showed that the total number of genes was 39, including 13 protein-coding genes, 22 tRNA genes, two rRNA genes, two O_H_ genes and 0 O_L_ genes. Amongst them were eight tRNA genes (trn-Q, trn-A, trn-N, trn-C, trn-Y, trn-P, trn-E and trn-S2) and the PCG gene nad6 on the main chain (J chain). The remaining 14 tRNA genes were trn-F, trn-V, trn-L2, trn-I, trn-M, trn-W, trn-D, trn-K, trn-G, trn-R, trn-H, trn-S1, trn-L1 and trn-T. Further, the two rRNA genes rrn-S and rrn-L were found and 12 PCGs genes encoding nad1, nad2, nad3, nad4, nad4L, nad5, atp6, atp8, cox1, cox2, cox3 and cytb on the secondary (N) chain were also found. There was no gene rearrangement (Fig. 1). The specific annotation results of each gene are shown in Suppl. material 1.

### Phylogenetic analysis

**Figure 2.**
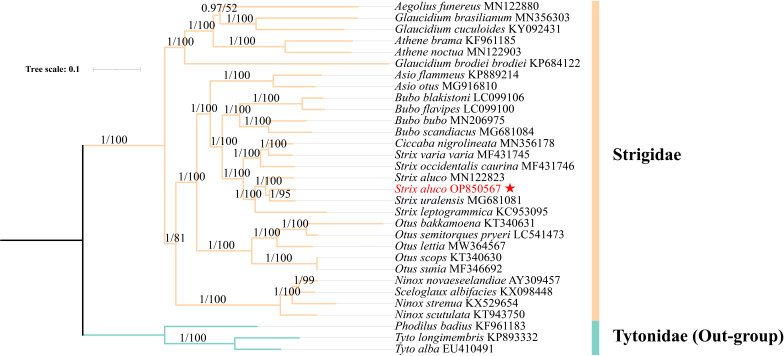
BI/ML-tree, Bayesian phylogenetic tree of 37 genes (24 rRNAs, 13PCGs) from 31 species of Strigiformes. The node labels are BI/ML posterior probability and bootstrap support value, respectively and the scale indicates the probability of nucleotide change within each branch length. The GenBank of the sequences has been indicated next to the species name. Branches of different subfamilies are distinguished by different colours, with Tytoninae (with *Phodilusbadius*, *Tytolongimembris* and *Tytoalba*) being the out-group. The *Strixaluco* mitochondrial genome obtained by this sequencing has been marked by ★.

In this study, both the ML-tree and BI-tree showed the same tree topology with good support. The tree showed that Strigidae and Tytonidae are two distinct lineages under the owl shape. *Athenenoctua* is a sister group of *Athenebrama*; *Glaucidium*, *Athene* and *Aegolius* constitute the same group, *Aegoliusfunereus* is closely related to (*G.cuculoides* + *G.brasilianum*), but BI/ML (posterior probability/bootstrap) is 0.97/52, the phylogenetic relationship between them is *Glaucidiumbrodieibrodiei* + ((*A.funereus* + (*G.cuculoides* + *G.brasilianum*)) + (*Athenenoctua* + *A.brama*). *Ciccabanigrolineata* is nested in *Strix*. It shows that BI/ML is 1/100, *S.aluco* in this study is a sister group of *S.uralensis*, *Strixaluco* MN122823 + (*Strixaluco* OP850567 + *Strixuralensis*) had formed with *S.aluco*; *Strix* is a sister to *Bubo* clade and forms an *Asio* + (*Strix* + *Bubo*) monophyletic group with *Asio* and a higher monophyletic group with *Otus* + [*Asio* + ((*Strix* + *Ciccabanigrolineata*) + *Bubo*)]. Additionally, *Sceloglauxalbifacies* is nested in *Ninox*, BI/ML is 1/99; this monophyly emerged simultaneously with (*Sceloglauxalbifacies* + *Ninox*) and the monophyly exhibited as dyadic taxa (Fig. 2).

### Divergence time evaluation

The divergence time tree, based on 37 genomes, shows that the time interval between Strigidae and Tytonidae from the common ancestor of Strigiformes was 8.05–12.75 Ma. However, in the out-group, *Tytoalba*, *Tytolongimembris* and *Phodilusbadius* diverged from the common ancestor at about 4.23–6.69 Ma. The divergence of Strigidae began at about 6.32–10.01 Ma, the common ancestor of Ninoxinae and Striginae in Strigidae split into two species at 5.50–8.7 Ma and the earliest divergence of Surniinae occurred in Strigidae, *Aegolius*, *Athene* and *Glaucidum* occurred at about 6.0–9.5 Ma, *Aegolius* diverged from the common ancestor of Surniinae during 5.10–8.08 Ma, *Athene* and *Glaucidium* diverged completely into two species during 4.82–7.64 Ma.

**Figure 3.**
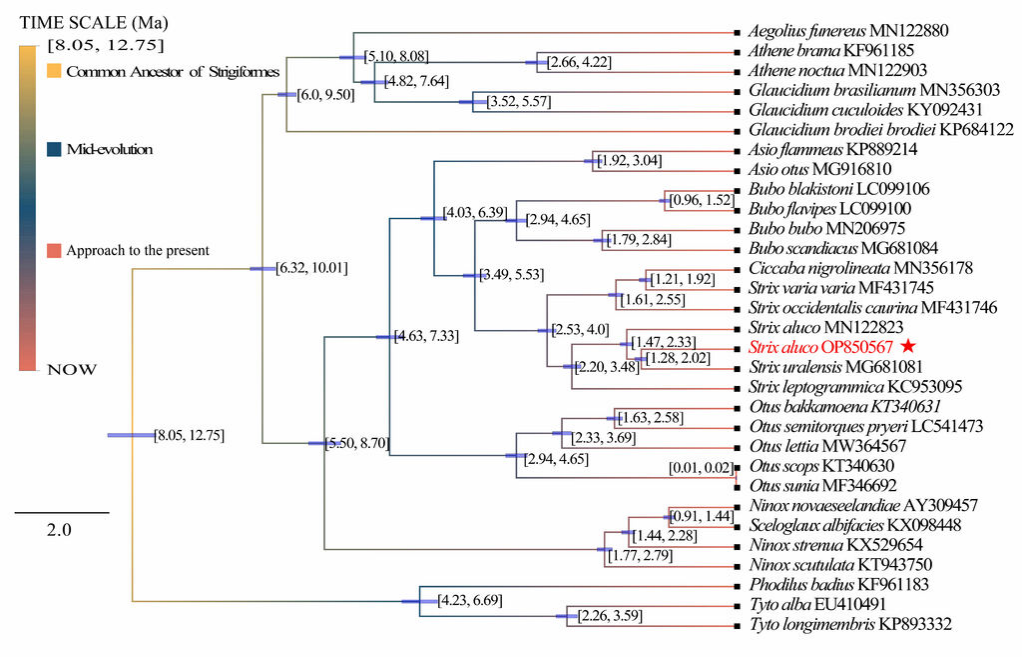
Divergence time tree. Through the divergence time tree obtained by BEAST 2.6.7, based on the Bayesian method, the node horizontal bar indicates that the posterior probability of this age interval is 95% and the divergence time has been marked at the node.

The common ancestor of *Strix* and *Bubo* diverged completely during 3.49–5.53 Ma and, during 2.53–4.0 Ma, *Strix* began to gradually diverge into multiple species. In this study, the divergence time between *S.aluco* (OP850567) and *S.aluco* of Margaryan. A (MN122823) was found to be about 1.47–2.33 Ma. The divergence time between *S.aluco* and *S.uralensis* in China was about 1.28–2.02 Ma (Fig. 3).

## Discussion

The mitochondrial genome structure of birds is a covalent double-chain loop structure, with a total of 37 genes, including 22 tRNAs, two rRNAs, 13 PCGs and 1–2 non-coding control regions (D-loop). The nad6 and eight tRNA encoding genes (trnQ, trnA, trnN, trnC, trnY, trnS2, trnP and trnE) are located on the J chain (light chain). The remaining 14 tRNAs, two rRNAs, 12 protein-coding genes and 1–2 non-coding control regions are all located on the N chain (heavy chain) (Wolstenholme 1992, Boore 1999), which is consistent with the complete mitochondrial genome structure of all birds (Hanna et al. 2017). The complete mitochondrial genome sequence of *S.aluco* obtained in this study was circular, with a total length of 18,632 bp and a GC content of 46.76%. Its composition was as follows: the ration of Adenine bases to the total base column (A%) was 29.57%; the ratio of the Guanine base to the total base column (G%) was 14.09%; the ratio of the Cytosine base to the total base column (C%) was 32.67%; the ratio of Thymidine to the total base column (T%) was 23.66%. The start codon of all 13 PCGs was ATG and the transcription stop codons were AGG, TAG and TAA. The content of A+T (53.23%) was higher than that of G+C (46.76%), which is consistent with the AT tendency of base bias in vertebrate mitochondrial genomes (Broughton et al. 2001, Ma et al. 2015). This result is consistent with the mitochondrial genome of other owls in Strigidae (Kang et al. 2018, Sun et al. 2020).

### Phylogenetic analysis of S.aluco

The BI and the ML tree have a consistent topology and each node has high posterior probability. The phylogenetic tree of Strigiformes obtained by the mitochondrial genome in this study is consistent with the phylogenetic tree obtained by *Li et al. (2022)* through morphology. Wink et al. (2009) compared Surniini (with Surnia, *Glaucidium* and *Taenioglaux*), Athenini (with *Athene*) and Aegolini (with *Aegolius*) under Surniinae, in their recent study, Surniini (with Surnia and *Glaucidium*) are monophyletic and cluster as a sister to *Aegolius* and they found that *Tytoalba* probably originated in Australia. They also believe that many owls that do not migrate will form new species in different places (Wink and Sauer-G&uuml 2021). Both Salter et al. (2020) and Wink and Sauer-G&uuml (2021) agree on the phylogenetic relationship of (*Glaucidium*+*Athene*) + *Aegolius* and we agree that *G.brodiei* does not form an evolutionary clade with other *Glaucidium*. According to the genome analysis of Strigidae birds in Madagascar, *Strix* is most closely related to *Bubo*, followed by *Otus* (Fuchs et al. 2008). The phylogenetic relationship of Strigidae forms a phylogenetic relationship of Surniinae (with Surniini and *Aegolius*) + [*Ninox* + (*Otus* + (*Asio* + (*Strix* + *Bubo*)))] in this study. The conclusion of *Otus* + (*Asio* + (*Strix + Bubo*)) is consistent with the conclusion of Kang et al. (2018). In the phylogenetic tree constructed by Yu et al. (2021), *C.nigrolineata* was also nested in *Strix*. *S.albifacies* has been extinct on the island of New Zealand and when Wood et al. (2017) extracted its mitochondrial genome from museum specimens, they suggested changing its name to *Ninoxalbifacies* because it has the same morphological structure and phylogenetic position as *Ninox*, in our phylogenetic tree, with relatively high posterior probability and bootstrap supporting this point. *S.aluco* OP850567 forms a sister group with *S.uralensis*, which was uploaded to GenBank by *Kang et al. (2018)*. However, *S.aluco* uploaded with Margaryan. A forms a monophyly of *Strixaluco* MN122823 + (*Strixaluco* OP850567 + *Strixuralensis*). Compared with the mitochondrial genome of *S.aluco* obtained by Margaryan. A, *S.aluco* in China is more closely related to *S.uralensis* MG681081, which came from northeast China. Extant Eurasian birds communicated through woodland corridors during the Pleistocene interglacial. Combined with divergence-time tree analysis, such communication may have existed in the common ancestor of S.aluco MN122823 and *S.aluco* OP850567 (Voelker 2010). The likely reason is that, at the beginning of the Pleistocene, the common ancestor of *S.aluco* MN122823 and *S.aluco* OP850567 had already been geographically isolated. The isolation of the Pleistocene refugium led to the divergence of the whole genome of the common ancestor of the forest owl both in China and internationally. Foreign studies have shown that the Quaternary Period is characterised by a series of glacial-interglacial cycles (*Woodruff 2010*), with the ancestors of modern species seeking refuge in suitable environments. The existing species on the Qinghai-Tibet Plateau may be the result of rapid population expansion in relatively warm refugia during the Pleistocene glaciation and interglacial period, which formed the current distribution pattern and genetic diversity (Gao et al. 2015). Leigong Mountain in Guizhou Province just played the role of a refugium for *S.aluco* during the Pleistocene glaciation period. Mitochondrial phylogeographic studies (Brito 2005) showed that the origin of *S.aluco* in Western Europe supports the "glacial refuge hypothesis"; further, the species survived in three allopatric refugees in Italy, the Iberian Peninsula and the Balkans, becoming the main source of *S.aluco* in Europe during the late glacial period. DNA barcoding technology also showed that the geographical barrier of the Strait of Gibraltar played an extremely important role in the phylogenetic history of *S.aluco* (Doña et al. 2016).

### Divergence time evaluation of S.aluco

The Pleistocene began 2.58 million years ago (2.58 Ma). The Pleistocene (especially climate change) had a profound effect on the phylogeographic structure of existing populations (Lamb et al. 2019). On the Qinghai-Tibet Plateau, the impact of mountain uplift on the formation of modern species (< 2.0 Ma) is limited and researchers have suggested that climate fluctuations played a key role in the formation of species during the Middle Pleistocene (Renner 2016, Wang et al. 2018). During the Quaternary Period and Pleistocene (1.60–2.70 Ma), there were severe climate shocks (Lisiecki and Raymo 2005), which positively promoted the formation of species (Rull 2008, Rull 2011, Rull 2015). Climatic fluctuations during this period, especially during the ice age, affected the distribution of forests in the Northern Hemisphere and the evolution of forest living species (Song et al. 2021). This series of climate fluctuations in the Pleistocene promoted species variation, which has led to species differentiation (Leonard et al. 2015). Glaciation has played an important role in influencing the population size, species and community genetic structure of today's species (Hewitt 2000, Hewitt 2004, Svendsen et al. 2004). The glacial-interglacial gyrations of the same period also affected the distribution of species (Zhao et al. 2013, Hung et al. 2014, Kozma et al. 2018, Mays et al. 2018). Glacial-interglacial cycles led to periodic shifts in glacial refuges for Pleistocene birds (*Nadachowska-Brzyska et al. 2015*) and the isolation of glacier refugia led to the divergence of the whole genome of species, thus forming different species (Provost et al. 2022). This is likely also the reason why the common ancestor of *S.aluco* MN122823 and *S.aluco* OP850567 (this study) diverged at 1.47–2.33 Ma. Genetic divergence of the same lineage because of the isolation of refugees leads to divergence of lineages. Various kinds of species generally begin to migrate to the best habitat during warm climate periods (Claramunt and Cracraft 2015); in particular, species adapted to low altitudes in the early stage of climate change will move to high altitudes at this time, resulting in the reproductive isolation of species in the two separated places (Wiens 2004). During the Pleistocene-Holocene (1.10–0.60 Ma), the Qinghai-Tibet Plateau experienced three stages of rapid uplift, with the formation of mountains, the climate changing from moist and warm to dry and cold and the retreat of forests to the edge of the plateau (*Wang et al. 2008*). Thus, the forest landscape became what it is today. The Quaternary Period climate shock led to the initial formation of the existing forest and mountain distribution pattern in the Northern Hemisphere. Birds began to distribute widely after leaving the glacier refuge at the end of the glaciation and initially formed the existing distribution pattern (Pujolar et al. 2022). The *S.uralensis* may have moved north at this time and thus diverged from *S.aluco*. In addition, the rapid uplift of the Qinling Mountains from the end of the Early Pleistocene to the Middle Pleistocene may have formed the Qinling Mountains as a barrier to north-south bird communication (Li et al. 2019). The rapid uplift of the Qinling Mountains prevented communication between *S.aluco* and the common ancestor of *S.uralensis*, which was originally distributed on the north and south sides.

## Conclusions

By sequencing the complete mitochondrial genome of *S.aluco* and mapping its phylogenetic tree and divergence time tree, the phylogenetic relationship of Strigiformes (Tytoninae + Phodilinae) + (Striginae + Ninoxinae + Surniinae) has been summarised. Tytonidae, including Tytoninae (with *Tyto*) and Phodilinae (with *Phodilus*), are defined as the out-group; Strigidae comprises Striginae (with *Asio*, *Bubo*, *Strix*, *Ciccaba* and *Otus*) + Ninoxinae + Surniinae (with *Athene*, *Aegolius* and *Glaucidium*). The divergence time tree shows that the divergence time between *S.aluco* of China and *S.aluco* of other countries was about 1.47–2.33 Ma, suggesting that the common ancestor of *S.aluco* was separated by geographical isolation at the beginning of the Pleistocene. The divergence between *S.aluco* and *S.uralensis* in China was about 1.28–2.02 Ma. During this time, the rapid uplift of the Qinling Mountains led to the divergence of the ancestors of *Strix* on the north and south sides of the Chinese mainland. At the same time, because of climatic oscillations during the Pleistocene, the existing *S.aluco* population on the Qinghai-Tibet Plateau may have rapidly expanded in relatively warm shelters, such as Leigong Mountain to form the current distribution pattern.

## Data ability

The complete mitochondrial genome of *Strixaluco* has been uploaded to NCBI, GenBank accession number: OP850567.

## Supplementary Material

BC9D0E06-5B18-50AC-BC8F-560FA9FD681F 10.3897/BDJ.11.e101942.suppl1 Supplementary material 1 Analysis of mitochondrial genome feature Data type Table Brief description The genome annotation results showed that the total number of genes was 39, including 13 protein-coding genes, 22 tRNA genes, two rRNA genes, two *O_H_* genes and 0 *O_L_* genes. Amongst them, eight tRNA genes (trn-Q, trn-A, trn-N, trn-C, trn-Y, trn-P, trn-E and trn-S2), one PCGs gene: nad6, are on the main chain (J chain); and the remaining 14 tRNA genes are trn-F, trn-V, trn-L2, trn-I, trn-M, trn-W, trn-D, trn-K, trn-G, trn-R, trn-H, trn-S1, trn-L1 and trn-T; Two rRNA genes: rrn-S, rrn-L;with 12 PCGs genes encoding: nad1, nad2, nad3, nad4, nad4L, nad5, atp6, atp8, cox1, cox2, cox3 and cytb on the secondary (N) chain. File: oo_818354.xlsx https://binary.pensoft.net/file/818354 Yeying Wang, Haofeng Zhan

## Figures and Tables

**Table 1.** Mitochondrial genome sequences used in this study.

**Taxon**	**GenBank accession**	**Size (bp)**	**Notes**	**Reference**
*Aegoliusfunereus*	MN122880	17166	Partial	Direct Submission
*Asioflammeus*	KP889214	18966	Complete	Zhang et al. (2016)
*Asiootus*	MG916810	17555	Complete	Lee et al. (2018)
*Athenebrama*	KF961185	16194	Partial	Direct Submission
*Athenenoctua*	MN122903	15776	Partial	Direct Submission
*Buboblakistoni*	LC099106	19379	Partial	Direct Submission
*Bubobubo*	MN206975	18956	Complete	Direct Submission
*Buboflavipes*	LC099100	19447	Partial	Direct Submission
*Buboscandiacus*	MG681084	18734	Complete	Kang et al. (2018)
*Ciccabanigrolineata*	MN356178	14875	Partial	Feng et al. (2020)
*Glaucidiumbrasilianum*	MN356303	17717	Partial	Feng et al. (2020)
*Glaucidiumbrodieibrodiei*	KP684122	17318	Complete	Sun et al. (2016)
*Glaucidiumcuculoides*	KY092431	17392	Complete	Liu et al. (2019)
*Ninoxnovaeseelandiae*	AY309457	16223	Complete	Harrison et al. (2004)
*Ninoxscutulata*	KT943750	16208	Complete	Direct Submission
*Ninoxstrenua*	KX529654	16206	Complete	Sarker et al. (2016)
*Otusbakkamoena*	KT340631	17389	Complete	Park et al. (2019a)
*Otuslettia*	MW364567	16951	Complete	Yu et al. (2021)
*Otusscops*	KT340630	17413	Complete	Park et al. (2019b)
*Otussemitorques*	LC541473	18834	Complete	Direct Submission
*Otussunia*	MF346692	17835	Complete	Zhou et al. (2019)
*Sceloglauxalbifacies*	KX098448	15565	Partial	Wood et al. (2017)
*Strixaluco*	MN122823	16490	Partial	Direct Submission
*Strixaluco*	OP850567	18832	Complete,	This study
*Strixleptogrammica*	KC953095	16307	Complete	Liu et al. (2014)
*Strixoccidentalis*	MF431746	19889	Complete	Hanna et al. (2017)
*Strixuralensis*	MG681081	18708	Complete	Kang et al. (2018)
*Strixvaria*	MF431745	18975	Complete	Hanna et al. (2017)
Out-group				
*Phodilusbadius*	KF961183	17086	Complete	Mahmood et al. (2014)
*Tytoalba*	EU410491	16148	Partial	Pratt et al. (2009)
*Tytolongimembris*	KP893332	18466	Partial	Xu et al. (2016)
